# Monocytic microRNA profile associated with coronary collateral artery function in chronic total occlusion patients

**DOI:** 10.1038/s41598-017-01695-3

**Published:** 2017-05-08

**Authors:** Nazanin Hakimzadeh, Joëlle Elias, Gilbert W. M. Wijntjens, Ruud Theunissen, Angela van Weert, Martijn W. Smulders, Nynke van den Akker, Perry D. Moerland, Hein J. Verberne, Loes P. Hoebers, Jose P. S. Henriques, Anja M. van der Laan, Mustafa Ilhan, Mark Post, Sebastiaan C. A. M. Bekkers, Jan J. Piek

**Affiliations:** 1 Department of Biomedical Engineering & Physics, Academic Medical Center, University of Amsterdam, Amsterdam, The Netherlands; 2 Department of Cardiology, Academic Medical Center, University of Amsterdam, Amsterdam, The Netherlands; 3 Department of Physiology, Maastricht University Medical Center, University of Maastricht, Maastricht, The Netherlands; 4 Cardiovascular Research Institute Maastricht (CARIM), Maastricht University, Maastricht, The Netherlands; 5 Department of Cardiology, Maastricht University Medical Center, University of Maastricht, Maastricht, The Netherlands; 6 Bioinformatics Laboratory, Academic Medical Center, University of Amsterdam, Amsterdam, The Netherlands; 7 Department of Nuclear Medicine, Academic Medical Center, University of Amsterdam, Amsterdam, The Netherlands

## Abstract

An expansive collateral artery network is correlated with improved survival in case of adverse cardiac episodes. We aimed to identify cellular microRNAs (miRNA; miR) important for collateral artery growth. Chronic total occlusion (CTO) patients (n = 26) were dichotomized using pressure-derived collateral flow index (CFI_p_) measurements; high collateral capacity (CFI_p_ > 0.39; n = 14) and low collateral (CFI_p_ < 0.39; n = 12) capacity. MiRNA profiling via next generation sequencing from various monocyte phenotypes (freshly isolated monocytes, monocytes cultured without stimulant, or stimulation with lipopolysaccharide, interleukin 4, transforming growth factor beta-1, or interferon gamma) revealed significantly different miRNA expression patterns between high versus low collateral capacity patients. Validation by real-time polymerase chain reaction demonstrated significantly decreased expression of miR339-5p in all stimulated monocyte phenotypes of low collateral capacity patients. MiR339-5p showed significant correlation with CFI_p_ values in stimulated monocytes. Ingenuity pathway analysis of predicted gene targets of miR339-5p and differential gene expression data from high versus low CFI_p_ patients (n = 20), revealed significant association with STAT3 pathway, and also suggested a possible regulatory role for this signaling pathway. These results identify a novel association between miR339-5p and coronary collateral function. Future work examining modulation of miR339-5p and downstream effects on the STAT3 pathway and subsequent collateral vessel growth are warranted.

## Introduction

Collateral arteries function as natural by-pass arteries, sustaining blood perfusion to tissue downstream of a coronary artery occlusion^1^. Transformation of pre-existing arteriolar anastomoses into mature collateral arteries is a form of vascular remodeling known as *arteriogenesis*. A well-developed collateral artery network can alleviate symptoms of ischemia, as well as preserve myocardial viability in case of adverse cardiac events^2–4^.

Circulating monocytes play a crucial role in collateral artery growth and maturation. Nonetheless, pharmacological intervention to promote arteriogenesis by enhancing monocyte survival and activity have led to disappointing outcomes in clinical trials^5–7^. Transcriptional profiling of circulating monocytes revealed heterogeneity at the messenger RNA (mRNA) level between coronary artery disease patients with insufficient versus sufficient coronary collateralization, whereby 244 genes were differentially expressed^8, 9^. Circulating monocytes from patients with low collateral capacity displayed active inhibitory pathways, in the form of heightened interferon-β and galectin-2 expression, preventing the growth of collateral arteries^8, 10^.

MicroRNAs (miRNA) have recently surfaced as novel targets for pharmaceutical development. These small non-coding RNAs (~22 nucleotides in length) regulate gene expression at a post-transcriptional level by impeding the translation or promoting degradation of downstream mRNA targets. We recently identified extracellular (plasma derived) circulating miRNAs differentially expressed in chronic total occlusion (CTO) patients with sufficient and insufficient collateral capacity^11^.

In this study we sought to unveil cellular miRNAs that may play a direct role in arteriogenesis. To do this we examined cellular miRNAs expressed in various monocyte/macrophage phenotypes in CTO patients with sufficient versus insufficient collateral artery capacity. Monocytes were activated with various stimulants to resemble M1 and M2 macrophage phenotypes. Both M1 and M2 macrophage phenotypes have been linked to collateral vessel growth^12, 13^.

## Results

### Patient Characteristics

Twenty-six patients undergoing elective PCI of a CTO were recruited, with a mean age of 63 ± 9 years and 24 (92%) were male (Table 1). All patients underwent invasive CFI_p_ measurements, resulting in a mean CFI_p_ of 0.42 ± 0.16. Frequency distribution of CFI_p_ values is depicted in Supplemental Figure 1. Patients were dichotomized into two groups based on a CFI_p_ threshold of 0.39, which is the mean CFI_p_ of a large CTO patient cohort (n = 295)^14^. Patient characteristics were comparable in low (CFI_p_ < 0.39) and high (CFI_p_ > 0.39) collateral capacity patient groups, with the exception of statin usage being higher in low CFI_p_ patients.

**Table 1:** Patient Characteristics.

Characteristic	CFI_p_ < 0.39 (n = 12)	CFI_p_ > 0.39 (n = 14)	P-value
Collateral flow index, mean ± SD	0.29 ± 0.05	0.55 ± 0.12	<0.0001
Age (years), mean ± SD	62 ± 11	64 ± 7	0.58
Male gender, n (%)	11 (92)	13 (93)	1.00
BMI (kg/m^2^), mean ± SD	28.1 ± 2.94	28.2 ± 5.14	0.95
Coronary Risk Factors			
Hypertension, n (%)	7 (58)	11(79)	0.65
Family history of CAD, n (%)	5 (42)	6 (43)	1.00
Hypercholesterolaemia, n (%)	5 (42)	1 (7.1)	0.06
Current smoker, n (%)	2 (17)	4 (29)	0.65
Past smoker, n (%)	6 (50)	4 (29)	0.42
Target vessel			
LAD (%)	6 (50)	2 (14)	0.12
RCA (%)	3 (25)	8 (57)	0.12
RCX (%)	3 (25)	3 (21)	0.12
History of Angina	8 (67)	9 (64)	1.00
Medication			
Salicylates, n (%)	9 (75)	14 (100)	0.08
ACE-inhibitors/ARBs, n (%)	1 (8.3)	5 (36)	0.17
β-blockers, n (%)	12 (100)	13 (93)	1.00
Statins, n (%)	11 (92)	1 (7.1)	<0.0001
Clopidogrel, n (%)	3 (25)	3 (21)	1.00
Calcium Antagonists, n (%)	4 (33)	5 (36)	1.00
Nitrates, n (%)	5 (42)	3 (21)	0.40
Laboratory values			
Hb (mmol/L)	9.09 ± 0.72	8.57 ± 1.07	0.18
RBC (10^12^/L)	4.92 ± 0.30	4.70 ± 0.44	0.17
WBC (10^9^/L)	7.67 ± 1.88	14.6 ± 23.9	0.35
Thrombocytes (10^9^/L)	249 ± 48.6	233 ± 45.2	0.42
Neutrophils (10^9^/L)	4.83 ± 1.46	5.11 ± 1.02	0.62
Eosinophils (10^9^/L)	0.189 ± 0.090	0.181 ± 0.092	0.84
Lymphocytes (10^9^/L)	2.01 ± 0.79	1.98 ± 0.62	0.93
Monocytes (10^9^/L)	0.66 ± 0.15	0.67 ± 0.19	0.90

ACE, Angiotensin converting enzyme; ARBs, angiotensin receptor blockers; BMI, body mass index; CAD, coronary artery disease; CFI_p_, collateral flow index; Hb, hemoglobin; LAD, left anterior descending; RBC, red blood cells; RCA, right coronary artery; RCX, right circumflex; WBC, white blood cell.

### NGS Results

**Figure 1:**
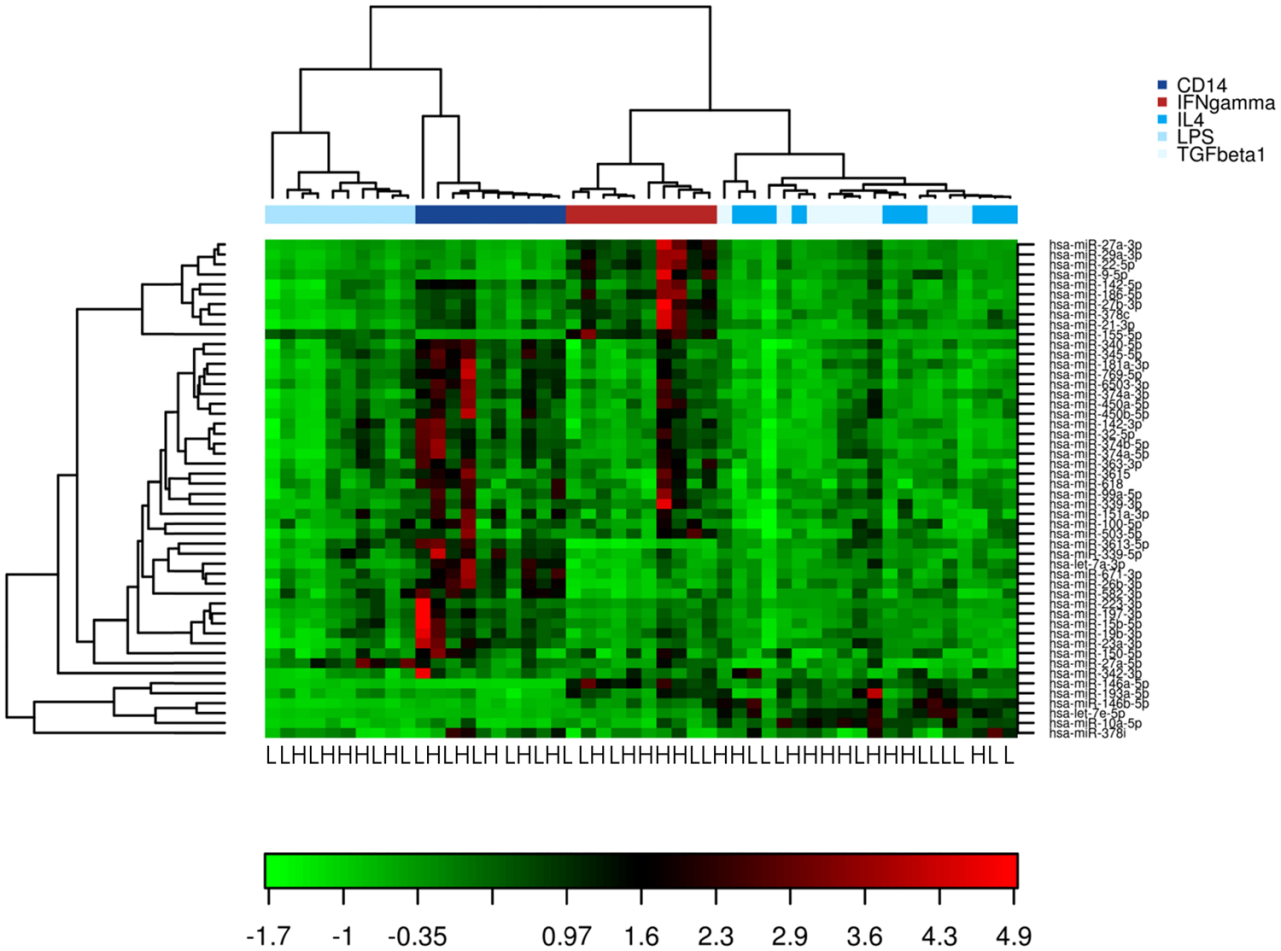
Differential microRNA expression in patients with high and low collateral capacity. Heat map and un-supervised hierarchical clustering of the top 50 microRNAs with the highest coefficient of variation (based on log2-transformed tag per million normalized data) in monocytes/macrophages of patients with high (n = 5) or low (n = 5) capacity of the collateral circulation. Each row represents one microRNA and each column represents one sample. The color scale shows the relative expression level of microRNA across samples, where red color depicts an expression level above mean and green color represents down regulated expression. H: high collateral capacity; L: Low collateral capacity.

To identify miRNAs that may be involved in collateral vessel development, we examined differential miRNA expression in patients with high and low collateral vessel capacity. Collateral vessel capacity was determined by CFI_p_ measurement. MiRNA isolated from each cell group of a subset of 10 patients (n = 5 high CFI_p_, and n = 5 low CFI_p_, patient characteristics shown in Supplemental Table 1) was profiled using NGS analysis to identify differential miRNA expression (schematically shown in Supplemental Figure 2). Patients were selected based on matching age, gender, medication usage and having CFI_p_ values from both ends of the spectrum. Unsupervised clustering of the top 50 miRNAs with highest coefficient of variation showed that samples group predominantly based on monocyte/macrophage phenotype (Fig. 1). Only M2 phenotypes, IL4 and TGFβ1, showed some overlapping miRNA expression patterns. MiRNA profiling further identified a number of miRNAs showing differential expression within each cell group between high and low collateral capacity patients (Supplemental Figure 3). These results are summarized in Supplemental Table 2.

### Validation by qPCR

MiRNAs demonstrating differential expression in at least two or more monocyte/macrophage phenotypes based on NGS results were selected for qPCR validation in the entire patient cohort (Supplemental Table 3). We also sought to examine the expression levels of miR126-5p and miR155-5p as these miRNAs showed differential expression in IL4 and IFNγ phenotypes respectively. MiR155-5p has been previously linked to arteriogenesis^15^, while miR126-5p has been shown to limit atherosclerosis by promoting endothelial turnover^16^. The Janus Phenomenon highlights that many atherogenic compounds are also pro-arteriogenic, while anti-atherogenic compounds are often anti-arteriogenic^5, 17^.

**Figure 2:**
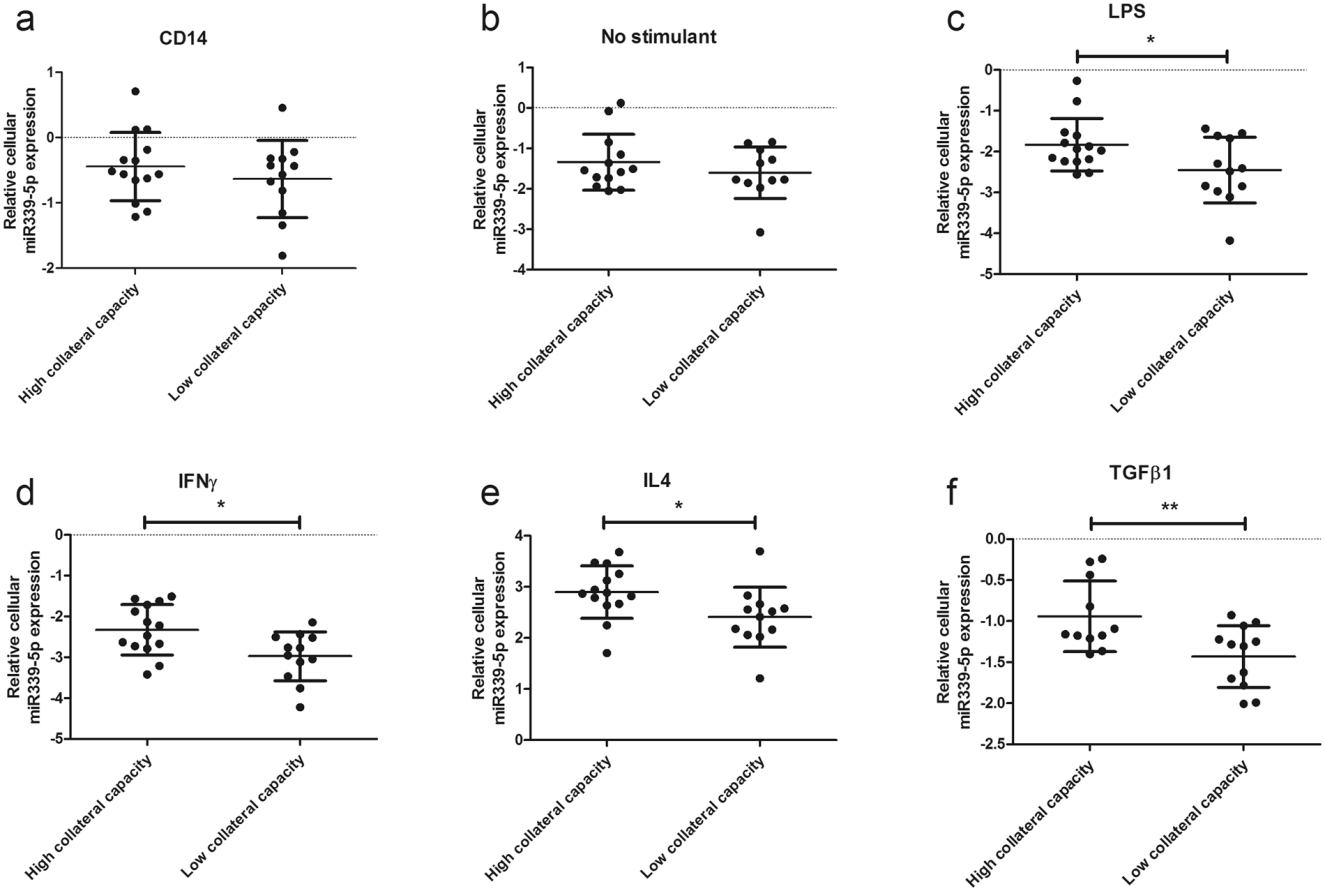
Relative cellular miR339-5p expression is lower in patients with low collateral capacity. Quantitative polymerase chain reaction measurements of miR339-5p in monocyte/macrophages of the total patient cohort (n = 26) with high versus low collateral capacity. (**a**) Freshly isolated CD14 monocyte, (**b**) monocyte cultured without stimulant or monocytes cultured with stimulant (**c**: LPS, **d**: IFNɣ, **e**: IL4, **f**: TGFβ1). Data are presented as mean ± SD. LPS: Lipopolysaccharide; IFNɣ: Interferon gamma; IL4: Interleukin 4; TGFβ1: Transforming growth factor beta 1.

**Figure 3:**
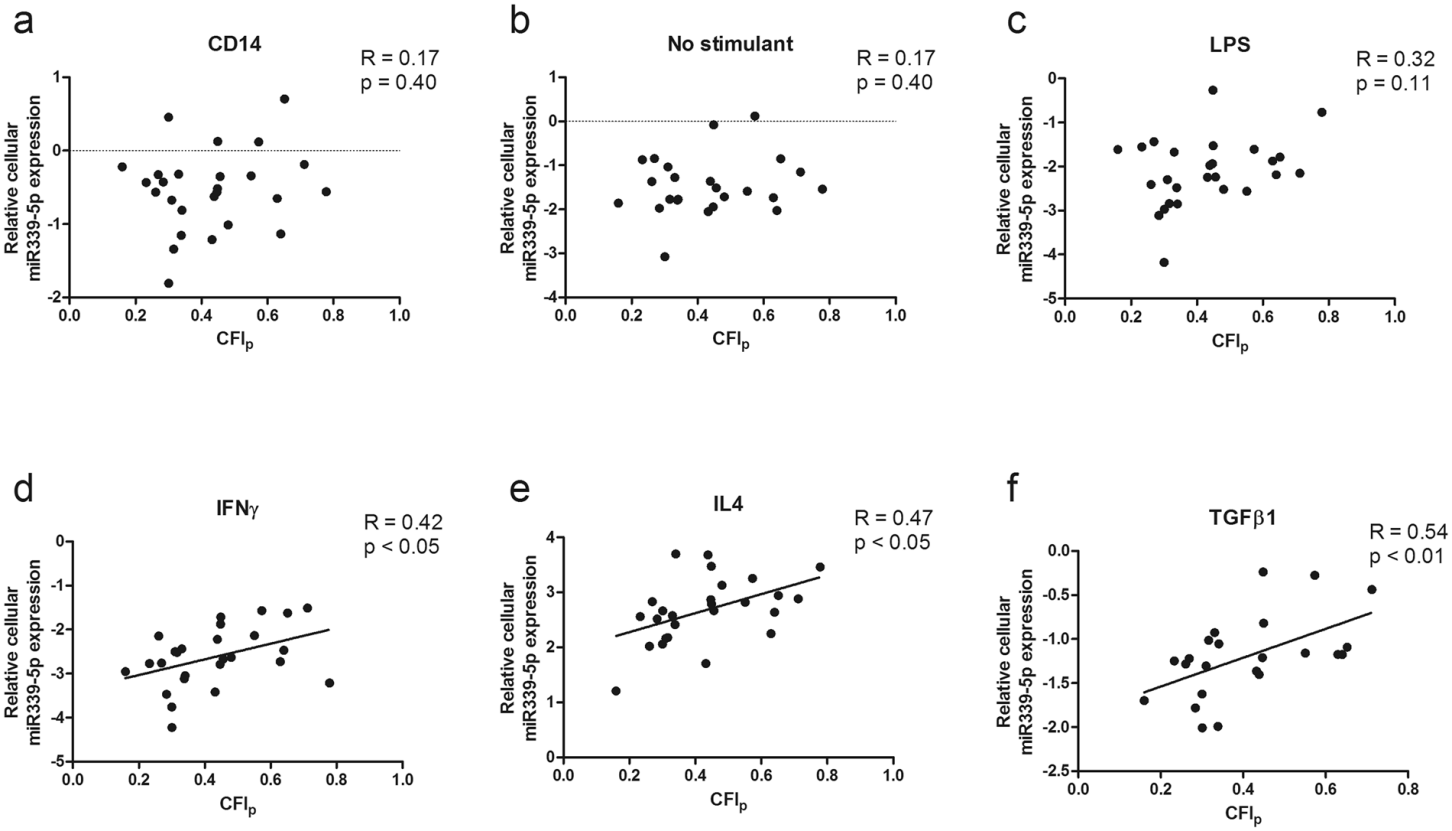
Correlation between CFI_p_ and relative miR339-5p expression in each monocyte/macrophage group. Relative miR339-5p is significantly correlated with CFI_p_ in certain monocyte/macrophage phenotypes (IFNɣ, IL4, TGFβ1).

Validation experiments using real-time RT–PCR analysis revealed that only miR339-5p demonstrated significant differential expression across multiple monocyte/macrophage phenotypes in patients with high versus low collateral capacity (Fig. 2). MiR339-5p showed significantly lower expression in all stimulated monocyte/macrophage phenotypes of patients with low collateral capacity, and not in freshly isolated CD14 monocytes or monocytes cultured without a stimulant. Relative miR339-5p expression was also significantly correlated with CFI_p_ in IFNγ, IL4 and TGFβ1 monocyte/macrophage stimulation groups (Fig. 3). Differential expression of miR339-5p was not seen in the supernatant of the different monocyte/macrophage phenotypes between high and low collateral capacity patients (Supplemental Figure 3). No other significant differences were seen in relation to cellular expression levels of the selected miRNAs between varying collateral capacity patients, with the exception of miR30b-5p in IFNγ stimulated monocytes. MiR30b-5p showed significantly lower expression in IFNγ stimulated monocyte/macrophages of low collateral capacity patients (Supplemental Figure 4).

**Figure 4:**
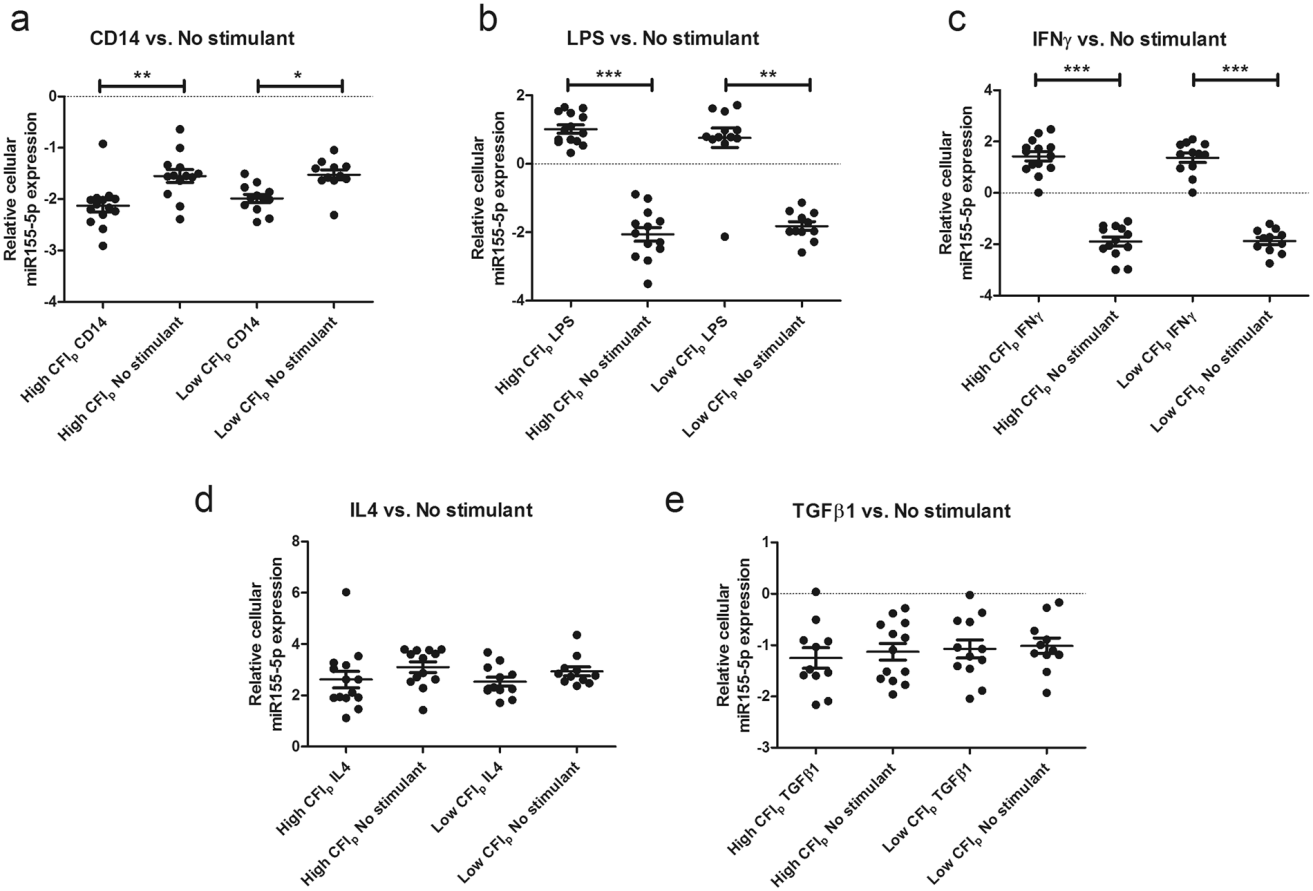
Relative cellular miR155-5p expression is higher in M1 stimulation phenotypes. To examine the effects of monocyte/macrophage stimulation, we compared relative cellular miR155-5p expression levels in the various monocyte/macrophage groups to monocytes cultured without stimulant (A: CD14 vs. No stimulant; B: LPS vs. No stimulant; C: IFNɣ vs. No stimulant; D: IL4 vs. No stimulant; E: TGFβ1 vs. No stimulant). Data presented are based on quantitative polymerase chain reaction measurements of miR155-5p in monocyte/macrophages of the total patient cohort (n = 26) with high (CFI_p_ > 0.39) or low (CFI_p_ < 0.39) collateral capacity. Data are presented as mean ± SD. *p < 0.05, **p < 0.01, ***p < 0.001. CFI_p_: Collateral flow index; LPS: Lipopolysaccharide; IFNɣ: Interferon gamma; IL4: Interleukin 4; TGFβ1: Transforming growth factor beta 1.

We also examined the effects of stimulation on miRNA expression, and noted significantly higher miR155-5p expression in monocyte/macrophages cultured without a stimulant relative to freshly isolated monocytes (Fig. 4). M1 monocyte/macrophage stimulation groups also showed increased miR155-5p expression than their non-stimulated counterparts. This upregulation was consistent in both high and low collateral capacity groups, however no significant difference was seen between monocyte/macrophages from patients with varying degrees of collateralization. Upregulation of miR155-5p was not seen in M2 monocyte/macrophage groups relative to cells cultured without stimulant.

### Ingenuity pathway analysis

IPA was utilized to identify enriched molecular networks, cellular functions as well as canonical pathways for miR339-5p (Table 2). Molecular and cellular functions associated with predicted targets of miR339-5p included cellular growth and proliferation, cellular assembly and organization, along with cellular function and maintenance. Pathway analysis suggested a significant association between predicted targets of miR339-5p and only two canonical pathways, Tumoricidal Function of Hepatic Natural Killer Cells and STAT3 pathway. VEGF Family Ligand-Receptor Interactions and Inhibition of Matrix Metalloproteases were also among the top associated canonical pathways, although the association was not significant.

**Table 2:** Top enriched canonical pathways, molecular as well as cellular functions of miR339-5p based on Ingenuity Pathway Analysis.

Canonical Pathways	p-value	Overlap
Tumoricidal Function of Hepatic Natural Killer Cells	2.21E-02	8.3% (2/24)
STAT3 Pathway	3.33E-02	4.1% (3/73)
Hippo signaling	5.01E-02	3.5% (3/86)
VEGF Family Ligand-Receptor Interactions	5.30E-02	3.4% (3/88)
Inhibition of Matrix Metalloproteases	5.40E-02	5/1% (2/39)
**Molecular and Cellular Functions**	**p-value**	**#Molecules**
Cellular Development	1.91E-02-3.35E-05	77
Cellular Growth and Proliferation	1.91E-02-3.35E-05	88
Cell Morphology	1.91E-02-1.28E-04	45
Cellular Assembly and Organization	1.91E-02-1.28E-04	55
Cellular Function and Maintenance	1.91E-02-1.28E-04	59

STAT3: Signal transducer and activator of transcription 3; VEGF: Vascular endothelial growth factor. P–value range for molecular and cellular functions represents the range of p-values generated for each subcategory and the respective molecules within each subcategory.

As predicted gene targets of miR339-5p were based on computational analysis and deductions, we cannot conclude upregulation or downregulation of these gene targets with this information alone. In order to understand how miR339-5p may regulate downstream gene expression, we examined mRNA expression data from various monocyte/macrophage phenotypes of high versus low collateral capacity CTO patients and looked for common associations with miR339-5p^10^. MiR339-5p was not a significant upstream regulator of gene expression data from the various monocyte/macrophage groups of high versus low collateral capacity. The STAT3 pathway showed significant association with differential gene expression data (high versus low collateral capacity CTO patients) from monocytes cultured without a stimulant, along with LPS and IL4 stimulation groups (Table 3). The identified target molecules represent molecules from the STAT3 canonical pathway, which match and are significantly associated with the respective gene sets. Although the target molecules associated with miR339-5p and CFI_p_ transcripts were not common, three target molecules were part of the same parent node, growth factor (GF) receptor family (Table 3 and Supplemental Figure 5). Furthermore, STAT3 was identified as a significant upstream regulator of LPS (p = 4.4E-11) and IL4 (p = 2.26E-6) stimulation gene sets and not an upstream regulator of freshly isolated monocytes or monocytes cultured without a stimulant. Meanwhile, miR155-5p was a significant upstream regulator of freshly isolated CD14 and IL4 stimulation gene sets (Supplemental Table 5). The gene sets examined consisted of all differentially expressed genes between high and low collateral capacity CTO patients in various monocyte/macrophage phenotypes^10^.

**Table 3:** Association between the STAT3 pathway and respective gene sets (miR339-5p predicted gene targets, or genes differentially expressed between high and low collateral capacity patients in monocytes cultured with no stimulant, or stimulation with LPS or IL4).

Gene set	p-value	Target molecules
miR339-5p predicted gene targets	p < 0.05	MAP3K10, FLT1, CDC25A
Monocytes cultured for 20 h without stimulant	p < 0.0001	IGF2R
LPS	p < 0.0001	TGFBR2
IL4	p < 0.001	PTPN2

The listed target molecules are molecules that are linked to each respective gene-set and are associated with the STAT3 pathway. High-lighted molecules share a common parent node in the STAT3 pathway; the common parent node being growth factor receptor (GF receptor).CDC25A, Cell division cycle 25 homolog A; FLT1, FMS related tyrosine kinase 1; IGF2R, Insulin-like growth factor 2 receptor; TGFBR2, Transforming growth factor beta receptor 2.

## Discussion

The present study demonstrates that stimulation of monocytes towards M1 and M2 phenotypes revealed lower expression of miR339-5p in low collateral capacity patients relative to high collateral capacity patients. Differential expression of miR339-5p was not seen in the supernatant and only at the cellular level. Relative expression of miR339-5p was also correlated with CFI_p_ values. Pathway analysis using IPA revealed molecular and cellular functions associated with miR339-5p. These functions included cellular growth and proliferation, cellular assembly and organization, as well as cellular function and maintenance. Comparison of differential mRNA expression data from high and low collateral capacity patients with predicted gene targets of miR339-5p revealed a common association with the STAT3 pathway. This pathway appears to be an upstream regulator of differentially expressed gene sets that are derived from stimulated monocyte phenotypes of high versus low collateralization.

In previous studies, transcriptional profiling of circulating monocytes revealed 244 differentially expressed genes in patients with insufficient versus sufficient collateralization^8–10^. Activation of inhibitory pathways, according to increased interferon-β^8^ and galectin-2^10^ mRNA expression in peripheral blood monocytes, impedes the growth of collateral vessels. Genetic heterogeneity in patients with varying degrees of collateralization was further shown with differential circulating miRNA expression in chronic total occlusion (CTO) patients with high versus low collateral capacity^11^. Circulating miR423-5p, miR10b, miR30d and miR126 have shown elevated expression levels in patients with insufficient collateralization^11, 18^.

According to these previous revelations, we hypothesized that patients with insufficient versus sufficient collateralization also display differential cellular miRNA expression levels. In this study we noted significantly lower levels of miR339-5p in stimulated monocytes derived from patients with low collateral capacity. We also noted lower relative expression of miR30b-5p in IFNɣ stimulated monocyte/macrophages from low collateral capacity patients. Dichotomization of patients into high and low collateral capacity groups was based on the mean CFI_p_ of the largest CTO patient group to date. CTO patients display no variability in lesion severity compared to coronary artery disease patients, and also display a higher mean CFI_p_ value than coronary artery disease patients. Relative expression of miR339-5p was also significantly correlated with CFI_p_ values in stimulated monocyte/macrophage phenotypes. Previous studies have implicated a role for miR339-5p in modulating cellular migration and invasion in metastatic cells, whereby its overexpression led to the inhibition of tumor-cell migration and invasion^19^. Furthermore, differential expression of miR339-5p was only noted in stimulated groups of monocyte/macrophages. This is consistent with the comparative pathway analysis results, whereby STAT3 was found to be an upstream regulator of gene sets derived from stimulated monocyte/macrophage phenotypes and not unstimulated cells. Collectively, this highlights the distinct signaling pathways that arise during macrophage polarization. We also noted elevated levels of miR155-5p in M1 macrophage phenotypes relative to unstimulated monocytes, however this difference was not noted between high and low collateralization groups. Heightened expression of miR155-5p is consistent with previous findings from Graff *et al*.^20^, whereby M1 polarized macrophages show greater levels of miR155-5p than unstimulated or M2 phenotypes. Interestingly, pathway analysis also revealed miR155-5p as a significant regulator of genes differentially expressed in freshly isolated monocytes and IL4 stimulated monocytes.

Differential expression of miR339-5p was not seen in the extracellular space and was only noted at the cellular level. This suggests a cell intrinsic role for the miRNA, particularly in polarized macrophage phenotypes. Previous studies have shown upregulation of miR339-5p following ischemic insults, whereby differential expression of miR339-5p was shown between various regions of ischemic area in the brain^21^. MiR339-5p showed upregulation in potentially salvageable apoptotic penumbra and not in necrotic tissue^21^. Furthermore, miR339-5p was also shown to be upregulated in response to human left ventricular ischemia^22^. Collectively, these studies suggest that this miRNA may have an important role in tissue remodeling. Thus, we can speculate that patients with insufficient collateralization may have limited vascular remodeling capacity due to lower levels of miR339-5p expression in their polarized macrophages.

Although miR339-5p was not a significant upstream regulator of gene sets differentially expressed between high and low collateralization, it was significantly associated with the STAT3 pathway. STAT3 was indeed an upstream regulator of differentially expressed gene sets in stimulated macrophage phenotypes of high and low collateralization. The JAK/STAT pathway is a principal signaling pathway involving over 70 cytokines, responsible for the initiation and constriction of innate and adaptive immune responses^23–26^. STAT3 is one of the members of the Signal Transducers and Activators of Transcription family. In macrophages, STAT3 has been shown to play an important role in limiting TLR signaling along with the inflammatory damage associated with M1 macrophage activation^27^. The STAT3 pathway has also been implicated in VEGF production as well as a facilitator of angiogenesis^28, 29^. Activation of this signaling pathway is required for endothelial cell migration and microvascular tube formation^30^. STAT3 pathway signaling pathway is also involved in the modulation the interferon signaling pathway^31, 32^, which has been shown to also regulate arteriogenesis^8, 9^. Thus, it is plausible that aberrant miR339-5p expression may lead to changes in STAT3 activation which ultimately affect collateral vessel formation. Alternatively, aberrant miR339-5p expression may be a by-product of divergent STAT3 pathway signaling in patients with high versus low collateral capacity. Nonetheless, these are speculations and must be confirmed by experimental validation. To date we are aware of elevation of Type I interferon signaling in stimulated monocytes of low collateral capacity patients^8, 9^, however, how this may be related to miR339-5p or STAT3 signaling remains to be realized.

### Study Limitations

We are limited by a significant difference in statin usage between high and low collateral capacity patients due to a low number of patients (n = 26). The imbalance in statin usage between high and low CFI_p_ patients may influence miRNA expression and collateralization. As a result, future studies with a larger patient cohort are required to examine causality. Nonetheless, our patient cohort consists of a wide range of CFI_p_ values with a comparable number of patients in high and low CFI_p_ groups. In addition, the significant differences seen are more powerful due to the multicenter nature of the study. Furthermore, when CFI_p_ is used as a continuous variable and thereby patients are not dichotomized into high and low collateral capacity groups, we indeed see a correlation between relative miR339-5p expression and CFI_p_ in stimulated monocyte groups. This reaffirms that the differences in miRNA expression are not attributable to differences in statin usage between high and low collateral capacity patients. Although we have identified an association between miR339-5p and coronary collateral artery capacity, we cannot yet confirm if this miRNA is directly involved in collateral vessel development. Furthermore, we have examined key biological processes along with cellular and molecular functions based on algorithm generated predicted targets of miR339-5p. We further compared these targets to mRNA expression data to identify predicted regulators. The predicted roles, associations and identification of STAT3 as a possible regulator are based on bioinformatics deductions which cannot replace experimental validation. Instead these results provide the basis for future studies investigating the functional consequences of miR339-5p on the STAT3 pathway and subsequently on collateral artery development.

### Conclusions

In conclusion, differential expression of cellular miRNAs in patients with high versus low collateral capacity is apparent. While many of these miRNAs are dependent on macrophage phenotype, miR339-5p demonstrated lower expression across multiple macrophage phenotypes in patients with low collateral capacity. Significant association with the STAT3 pathway between miR339-5p and mRNA based gene expression data suggests a possible modulatory role for the STAT3 pathway in collateral vessel development. Future work examining inhibition of miR339-5p expression and the effects on STAT3 pathway along with collateral artery development are imperative.

## Methods

Expanded methods section is provided in the supplemental material.

### Patient population and procedures

This study was conducted in accordance with the Declaration of Helsinki. The institutional medical ethics committee of the Academic Medical Center of the University of Amsterdam and Maastricht University Medical Center approved the study protocol. All patients gave written informed consent. Briefly, 26 patients that underwent successful elective percutaneous coronary intervention (PCI) of a CTO were included. Patients were considered eligible if they had symptoms of angina pectoris for ≥4 weeks and a CTO of a coronary artery. Exclusion criteria included previous transmural myocardial infarction in the area of the CTO based on electrocardiographic (ECG) assessment. Furthermore, cardiac surgery, depressed left ventricular function (ejection fraction < 30%) and inflammatory or neoplastic disease were also considered as exclusion criteria. Pressure-derived collateral flow index (CFI_p_) was measured as described previously^9–11^. Fifteen patients were recruited at the Academic Medical Center and 11 patients were included at the Maastricht University Medical Center. Patients were dichotomized into two groups based on their CFI_p_ value, whereby CFI_p_ > 0.39 were considered high collateral capacity and CFI_p_ < 0.39 were deemed low collateral capacity. This CFI_p_ value was selected based on a previous study showing that 0.39 was the mean CFI_p_ value in a large CTO patient cohort (n = 295)^14^.

From each patient arterial blood was withdrawn immediately before PCI and ethylenediaminetetraactic acid (EDTA) coagulated to attain six monocytic cell phenotypes. Peripheral blood mononuclear cells were initially isolated by density gradient centrifugation using Lymphoprep (Stemcell Technologies). Unstimulated CD14+ monocytes were positively isolated using anti-CD14-coated immunomagnetic beads (Magnetic-activated cell sorting, MACS; Miltenyi biotec). CD14+ monocyte purity was measured by flow cytometry (BD FACS Canto II with FACSDiva Version 6.1.3 or Beckman Coulter FC500 with Beckman Coulter Kaluza software), after probing with monoclonal mouse anti-human CD14-FITC (Abd Serotec). Isolated CD14+ monocytes were 96.0 ± 1.5% pure. CD14+ monocytes were either cultured for 3 hours (h) in the presence of 10 ng/mL lipopolysaccharide (LPS; Sigma), or no stimulant. Three other monocyte phenotypes were also generated by overnight culture of CD14+ monocytes in the presence of 20 ng/mL interleukin (IL)-4 (Sigma), 20 ng/mL interferon ɣ (IFNɣ; R & D Systems), or 0.5 ng/mL transforming growth factor β1 (TGFβ1; Peprotech). Freshly isolated CD14+ cells were snap-frozen and stored at −80 °C until RNA isolation. After culture, all adherent and non-adherent cells were lysed and total RNA was isolated using Exiqon miRCury RNA isolation kit – Cell & Plant kit (Exiqon) according to the manufacturer’s instructions. Supernatant from each cell group was stored at −80 °C until RNA isolation.

### Next Generation Sequencing

The following steps were conducted by Exiqon Services, Denmark. Total RNA was converted into miRNA next generation sequencing (NGS) libraries using NEBNEXT library generation kit (New England Biolabs Inc.) using the manufacturer’s instructions.

### Validation of next generation sequencing results by real-time PCR

Validation of NGS results by real-time PCR was conducted on the entire patient cohort. RNA was reverse transcribed into cDNA using the miRCURY LNA™ Universal RTmicroRNA PCR, Polyadenylation and cDNA synthesis kit (Exiqon) according to the manufacturer’s instructions. CDNA was assayed in PCR reactions according to the manufacturer’s protocol of miRCURY LNA™ Universal RT microRNA PCR. Normalization was performed using stably expressed endogenous controls for each monocyte/macrophage phenotype (Supplemental Table). Stably expressed endogenous controls were pre-selected for each cell condition according to the criteria of having the lowest coefficient of variation between high and low collateral capacity patients. Due to the different stimulation conditions, there was a lack of one common normalizer appropriate for all cell phenotypes.

### Ingenuity Pathway Analysis (IPA)

Predicted gene targets of the validated differentially expressed miRNAs (miR339-5p and miR155-5p) were submitted to Ingenuity Pathway Analysis (Ingenuity Systems, http://www.ingenuity.com). We generated a list of predicted target genes for each select miRNA using TargetScan 7.1 (www.TargetScan.org)^33^. We found 207 predicted gene targets for miR339-5p and 552 predicted gene targets were found for miR155-5p. These predicted targets were entered into IPA software to identify key biological processes, canonical pathways, molecular functions and genetic networks that may be regulated by these miRNAs. Differential mRNA expression data from high versus low collateral capacity patients from various monocyte/macrophage phenotypes (unstimulated monocytes, LPS stimulated monocytes, IL-4 stimulated monocytes and monocytes cultured for 20 hours without stimulant) from a separate CTO patient cohort^10^ were also examined and compared to miR339-5p predicted targets to identify key canonical pathways and upstream regulators that may be activated or inhibited. In brief, gene set enrichment analysis was implemented to identify differentially expressed genes in various monocyte/macrophage phenotypes of patients with high and low CFI. Genes were deemed to be differentially expressed if the Bayesian statistics t-test (Cyber-T) p-value was below 0.05, and the absolute fold change was greater than 1.2^10^. The core and comparative analysis tools of IPA software were utilized to examine data from all species. Within the core analysis of IPA, canonical pathway analysis associates gene sets input by the user (ie. predicted gene targets of miR339-5p, or CFI_p_ associated transcripts) with known gene sets in Ingenuity’s Knowledge Base. Two measures of association with known canonical pathways are generated, one being a ratio of the number of genes from the known list that map to the input gene set, and the second measure is a p-value of the Fisher’s exact test.

### Statistics

Student’s two-tailed t-test was used to compare data with a normal distribution between two groups. Data with a non-normal distribution were compared between two groups using the Mann-Whitney U-test. Analysis of variance was used if normally distributed data were compared between more than two groups, followed by Tukey’s post hoc test. The Kruskal-Wallis test was used to compare non-normally distributed data, followed by Dunns post-hoc test. Statistical differences in categorical data between groups were examined with Fisher’s exact test. The Chi-square test was utilized to look for statistical differences between the CTO target vessel in high versus low collateral capacity patients. Correlations were determined using a Pearson correlation. Statistical analysis was performed using GraphPad Prism 5 and IBM SPSS Statistics 20, whereby statistical significance was deemed when a p-value was less than 0.05.

## Electronic supplementary material

Supplementary Material
