# Supplementary material for: Monocytic microRNA profile associated with coronary collateral artery function in chronic total occlusion patients
Source: Sci Rep. 2017 May 8;7:1532. doi: 10.1038/s41598-017-01695-3 (PMC5431477; doi:10.1038/s41598-017-01695-3)
Supplement: Supplementary file 1 — Supplementary Material [file 41598_2017_1695_MOESM1_ESM.pdf]

Monocytic microRNA profile associated with coronary collateral artery function in chronic total occlusion patients

Nazanin Hakimzadeh, Joelle Elias, Gilbert W.M. Wijntjens, Ruud Theunissen, Angela van Weert, Martijn W. Smulders, Nynke van den Akker, Perry D. Moerland, Hein J. Verberne, Loes P. Hoebers, Jose P.S. Henriques, Anja M. van der Laan, Mustafa Ilhan, Mark Post, Sebastiaan C.A.M. Bekkers, Jan J. Piek

Supplementary material

Supplementary methods

RNA isolation from supernatant

Supernatants were thawed (on ice) and centrifuged (3000 g, 5 min, 4 ºC). An aliquot (200 µL) was transferred to a FluidX tube with 60 µL lysis solution BF containing carrier-RNA (1 µg/60 µL). RNA spike-in template mixture was also added to the sample, mixed and incubated at room temperature (7 min), after which 20 µL Protein precipitation solution BF was added. Samples were centrifuged and supernatants were subjected to Exiqon's internal proprietary bead based RNA isolation protocol. Purified RNA was eluted in 50 µL final volume and stored at -80 ºC until further processing.

Next Generation Sequencing

Each RNA sample had adaptors ligated to its 3' and 5' ends and subsequently converted into complementary deoxyribonucleic acid (cDNA). The cDNA was pre-amplified with specific primers containing sample specific indexes. After 15 cycle pre-PCR the libraries were purified on QiaQuick columns and the insert efficiency evaluated by Bioanalyzer 2100 instrument on high sensitivity DNA chip (Agilent Inc.). MiRNA cDNA libraries were size fractionated on a LabChip XT (Caliper Inc.) and a band representing adaptors and 15-40 bp insert was excised according to the manufacturer's instructions. Samples were then quantified using quantitative polymerase chain reaction (qPCR) and concentration standards. Based on quality of the inserts and the concentration measurements the libraries were pooled in equimolar concentrations. Library pools were quantified again with qPCR and optimal concentration of the library pool was used to generate the clusters on the surface of a flowcell. Sequencing was then conducted using NextSeq 500 sequencing methodology and reagents based on the manufacturer instructions (Illumina Inc.). On average 14.1 million 50-bp single-end reads were obtained per sample. Adapters were trimmed and subsequently reads representing high quality sequencing data were mapped to the human reference genome (GRCh37/hg19) annotated with miRBase version 20 to count reads associated with known human microRNAs. The number of miRNAs identified in all samples with at

least one tag per million (TPM) mapped reads was 262. Differential expression analysis was done using the EdgeR package in the statistical software environment R. Counts were normalized using the trimmed mean of M-values (TMM normalization) method[1], to compensate for sample specific effects caused by variation in library size/sequencing depth between samples. TMM normalization was conducted in the EdgeR statistical software package (Bioconductor, http://www.bioconductor.org/). For each miRNA and each phenotype P-values for differential expression between high and low CFIp patients were determined by an exact test on the negative binomial distribution.

Validation of next generation sequencing results by real-time PCR

ExiLENT SYBR® Green master mix was utilized to assay each selected miRNA once by qPCR on the microRNA Ready-to-Use PCR Custom Pick and Mix Panel. Negative controls excluding template from the reverse transcription reaction were included and assayed the same as samples. Amplification was conducted in a LightCycler® 480 Real-Time PCR System (Roche) with 384 well plates. Roche LC software was employed to assess amplification curves for calculation of the quantification cycle (Cq) values by the 2nd derivative method as well as melting curve examination. Normalization of cellular and extracellular data was performed using stably expressed endogenous controls for each monocyte/macrophage phenotype (Supplemental Figure 3). These controls were preselected based on the NGS results.

Supplementary material

Supplementary Table 1. Characteristics of patients used for next generation sequencing (NGS) analysis.

Characteristic	CFI_p <0.39 (n=5)	CFI_p >0.39 (n=5)	P-value
Collateral flow index, mean ± SD	0.25±0.06	0.63±0.13	p<0.001
Age (years), mean ± SD	62±6	60±14	0.71
Male gender, n (%)	5 (100)	5 (100)	1.00
BMI (kg/m^2), mean ± SD	28.6±3.14	30.1±3.25	0.48
Coronary Risk Factors			
Hypertension, n (%)	4 (80)	4 (80)	1.00
Family history of CAD, n (%)	3 (60)	2 (40)	1.00
Hypercholesterolaemia, n (%)	2 (40)	3 (60)	1.00
Current smoker, n (%)	0 (0)	2 (40)	0.44
Past smoker, n (%)	2 (40)	2 (40)	1.00
Target vessel			
LAD (%)	3 (60)	0 (0)	0.17
RCA (%)	1 (25)	4 (80)	0.21
RCX (%)	1 (25)	1 (25)	1.00
History of Angina, n (%)	4 (80)	3 (60)	1.00
Medication			
Salicylates, n (%)	5 (100)	5 (100)	1.00
ACE-inhibitors/ARBs, n (%)	1 (25)	2 (40)	1.00
β - blockers, n (%)	5 (100)	4 (80)	1.00
Statins, n (%)	4 (80)	5 (100)	1.00
Clopidogrel, n (%)	1 (25)	1 (25)	1.00
Calcium Antagonists, n (%)	3 (60)	2 (40)	1.00
Nitrates, n (%)	2 (40)	0 (0)	0.44

ACE, Angiotensin converting enzyme; ARBs, angiotensin receptor blockers; BMI, body mass index; CAD, coronary artery disease; CFI_p, collateral flow index; Hb, hemoglobin; LAD, left anterior descending; RBC, red blood cells; RCA, right coronary artery; RCX, right circumflex; WBC, white blood cell.

Supplementary Table 2. List of miRNAs differentially expressed in each cell condition in order of ascending p-value.

MiRNA per cell condition	logFC	p-value	FDR
**CD14**			
hsa-miR-151a-3p	-0.67	0.0012	0.38
hsa-miR-6718-5p	-0.64	0.0041	0.64
hsa-miR-342-3p	0.85	0.0098	0.77
hsa-miR-454-3p	0.54	0.0099	0.77
hsa-miR-125b-5p	-0.64	0.015	0.81
hsa-miR-3690	-1.23	0.016	0.81
hsa-miR-582-5p	0.76	0.019	0.82
hsa-miR-1278	-0.53	0.021	0.82
hsa-miR-4443	1.26	0.024	0.83
hsa-miR-1255a	0.89	0.028	0.87
hsa-miR-324-5p	0.67	0.038	0.92
hsa-miR-3607-3p	0.47	0.04	0.92
hsa-miR-29b-3p	0.42	0.045	0.92
hsa-miR-34c-5p	0.54	0.048	0.92
hsa-miR-339-3p	-0.4	0.052	0.92
hsa-miR-877-3p	0.55	0.052	0.92
**LPS**			
hsa-miR-486-3p	-1.26	0.00083	0.25
hsa-miR-92a-1-5p	0.89	0.0029	0.44
hsa-miR-193a-5p	0.54	0.0063	0.58
hsa-miR-3690	-1.28	0.0076	0.58
hsa-miR-1307-5p	-0.8	0.011	0.59
hsa-miR-30b-5p	-0.64	0.012	0.59
hsa-miR-450b-5p	-0.56	0.015	0.59
hsa-miR-339-5p	-0.64	0.015	0.59
hsa-miR-29b-1-5p	0.52	0.02	0.66
hsa-let-7i-3p	-0.58	0.023	0.68
hsa-miR-335-3p	-0.6	0.025	0.68
hsa-miR-1255a	0.78	0.028	0.7
hsa-miR-378a-5p	-0.4	0.034	0.75
hsa-miR-320c	-0.61	0.034	0.75
hsa-miR-451a	1.21	0.048	0.95
hsa-let-7f-2-3p	-0.51	0.052	0.95
**IFNγ1**			
hsa-miR-122-5p	-0.89	0.006	0.71
hsa-let-7i-3p	-0.95	0.0064	0.71
hsa-miR-1307-5p	-0.9	0.0082	0.71

hsa-miR-3607-3p	0.69	0.0091	0.71
hsa-miR-3690	-1.34	0.025	0.95
hsa-miR-345-5p	-0.42	0.031	0.95
hsa-let-7a-3p	-0.53	0.032	0.95
hsa-miR-223-5p	0.44	0.033	0.95
hsa-miR-155-5p	0.49	0.037	0.95
hsa-miR-486-3p	-0.81	0.038	0.95
hsa-miR-339-5p	-0.56	0.047	0.95
hsa-let-7f-1-3p	-0.67	0.055	0.95
**TGFβ1**			
hsa-miR-1248	0.88	0.0012	0.3
hsa-miR-320d	-0.85	0.002	0.3
hsa-miR-582-5p	0.78	0.0043	0.34
hsa-miR-4662a-5p	-0.87	0.005	0.34
hsa-miR-3690	-1.46	0.0056	0.34
hsa-miR-486-3p	-1.21	0.0073	0.34
hsa-miR-151a-3p	-0.49	0.0078	0.34
hsa-miR-1246	-0.64	0.011	0.42
hsa-miR-4791	-0.61	0.016	0.54
hsa-miR-320c	-0.57	0.024	0.65
hsa-miR-29b-2-5p	0.58	0.027	0.65
hsa-miR-511-5p	-0.81	0.027	0.65
hsa-miR-1307-5p	-0.57	0.027	0.65
hsa-miR-122-5p	-1.03	0.038	0.78
hsa-miR-1908-5p	-0.75	0.045	0.78
hsa-miR-3158-3p	-0.6	0.045	0.78
hsa-miR-218-5p	1.3	0.045	0.78
hsa-miR-3607-3p	0.61	0.046	0.78
**IL4**			
hsa-miR-3690	-1.38	0.012	0.84
hsa-miR-664a-5p	0.73	0.014	0.84
hsa-miR-18a-5p	-0.55	0.017	0.84
hsa-miR-374b-3p	-0.81	0.019	0.84
hsa-miR-335-5p	-0.56	0.021	0.84
hsa-miR-1255a	0.96	0.032	0.84
hsa-miR-30b-5p	-0.49	0.039	0.84
hsa-miR-126-5p	-0.63	0.045	0.84
hsa-miR-148a-5p	0.67	0.045	0.84
hsa-miR-223-5p	0.31	0.052	0.84

Numerous miRNAs demonstrated differential expression in the different monocyte/macrophage phenotypes between high and low CFI patients. Log fold change (logFC) is relative to low CFI. Therefore a negative value indicates decreased expression in monocyte/macrophages from low CFI patients in comparison to that of high CFI patients. All false discovery rate (FDR) values were above 0.05, thus none of the miRNAs pass multiple correction testing in the next generation sequencing (NGS) experiments. IFNγ: interferon gamma 1; IL4: interleukin 4; LPS: lipopolysaccharide; TGFβ1: transforming growth factor beta 1.

Supplemental Table 3: List of microRNAs (miRNA) selected for validation by quantitative polymerase chain reaction showed differential expression patterns in at least two or more monocyte/macrophage phenotypes in next generation sequencing experiments. Two additional miRNAs (miR126-5p and miR155-5p) were also selected for qPCR validation based on previous studies linking these miRNAs to collateral vessel growth. Refer to supplemental Table 1 for respective logFold Changes and p-values.

**miRNA**	**Monocyte/Macrophage Phenotypes with differential expression**	**Expression pattern**
miR151a-3p	CD14, TGFβ1	Decreased expression in low CFI
miR3607-3p	CD14, IFNγ, TGFβ1	Increased expression in low CFI
miR30b-5p	LPS, IL4	Decreased expression in low CFI
miR339-5p	LPS, IFNγ	Decreased expression in low CFI
miR122-5p	IFNγ, TGFβ1	Decreased expression in low CFI
miR1307-5p	LPS, IFNγ, TGFβ1	Decreased expression in low CFI
miR126-5p	IL4	Decreased expression in low CFI
miR155-5p	IFNγ	Decreased expression in low CFI

Supplemental Table 4: Coefficient of variation values for miRNAs selected as endogenous controls based on next generation sequencing (NGS) analysis. MiRNAs with the lowest coefficient of variation, highest p-value (high vs. low CFI) and sufficient expression levels were selected as the most suitable endogenous control for each monocyte/macrophage phenotype for qPCR validation experiments. Coefficient of variation values were calculated from for each miRNA within each cell type using NGS values from high and low CFI patients. Values highlighted in grey represent the miRNA selected to be used as an endogenous control for the respective cell condition.

	Coefficient of Variation					
**Cell condition**	**let-7f-5p**	**let-7g-5p**	**miR30d-5p**	**miR191-5p**	**miR221-3p**	**miR501-5p**
CD14	0.25	0.21	0.17	0.19	0.15	0.20
LPS	0.14	0.13	0.18	0.20	0.25	0.27
IFNγ	0.19	0.14	0.21	0.13	0.17	0.33
IL4	0.32	0.28	0.22	0.23	0.21	0.12
TGFβ1	0.16	0.21	0.12	0.16	0.20	0.28

Supplemental Table 5. MiR155 is an upstream regulator of differentially expressed genes in freshly isolated (CD14) and interleukin 4 (IL4) stimulated monocytes in patients with high versus low collateral capacity.

Gene set	p-value	Target Molecules
CD14	$p < 0.01$	IRAK3,TNPO3
IL4	$p < 0.05$	ANTXR2,CD274,CYP51A1,DCUN1D3,FCER2,HSD17B12, MAF,MYB,PTPN2,RGL1,SORL1

Supplemental Figure 1. Frequency distribution of collateral flow index (CFI_p) in patient cohort (n = 26). Patients were dichotomized into two groups based on low ($CFI_p < 0.39$) and high ($CFI_p > 0.39$) collateral capacity according to their CFI_p values.

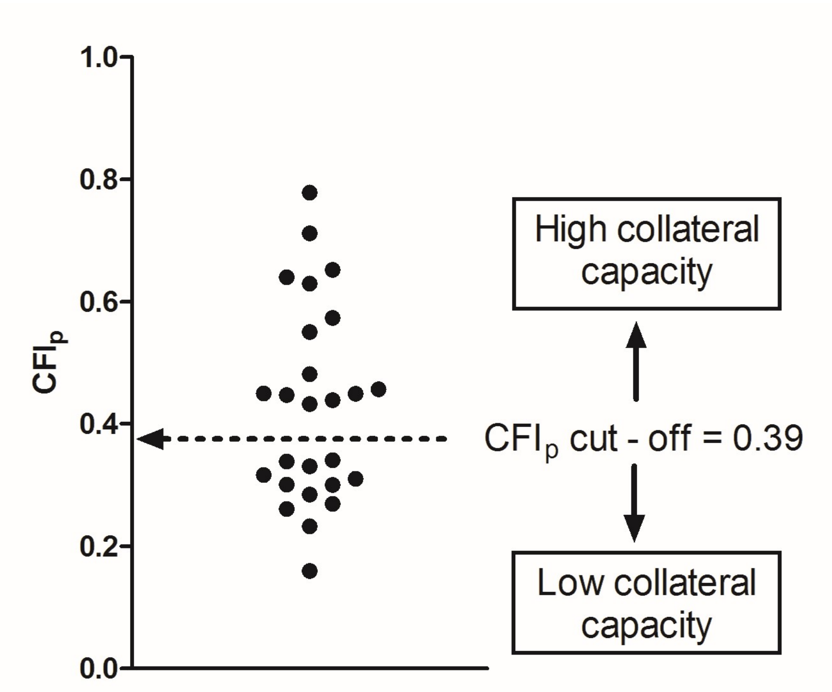

Supplemental Figure 2. Differential microRNA expression within each monocyte/macrophage phenotype between patients with high versus low collateral capacity. Heat-maps depict the top 50 microRNAs with the lowest p-value in monocytes/macrophages of patients with high (n=5) versus low (n=5) collateral capacity. (A) Freshly isolated CD14 monocytes or monocyte/macrophages cultured with stimulant (B: LPS, C: IFNɣ, D: IL4, E: TGFβ1). Each row represents one microRNA and each column represents one sample. The color scale shows the relative expression level of microRNA across samples, where red color depicts an expression level above mean, green color represents down regulated expression and grey color indicates undetectable expression. Within each heat-map, high CFI samples are clustered to the left of the heat-map (green panel), while low CFI samples are clustered to the right (blue panel). Horizontal dendogram axis represents the distance and in turn dissimilarity between clusters. Clustering was determined with Pearson correlation as distance metric and average linkage clustering. CFI: collateral flow index; IFNɣ: Interferon gamma; IL4: Interleukin 4; LPS: Lipopolysaccharide; TGFβ1: Transforming growth factor beta 1.

Supplemental Figure 2

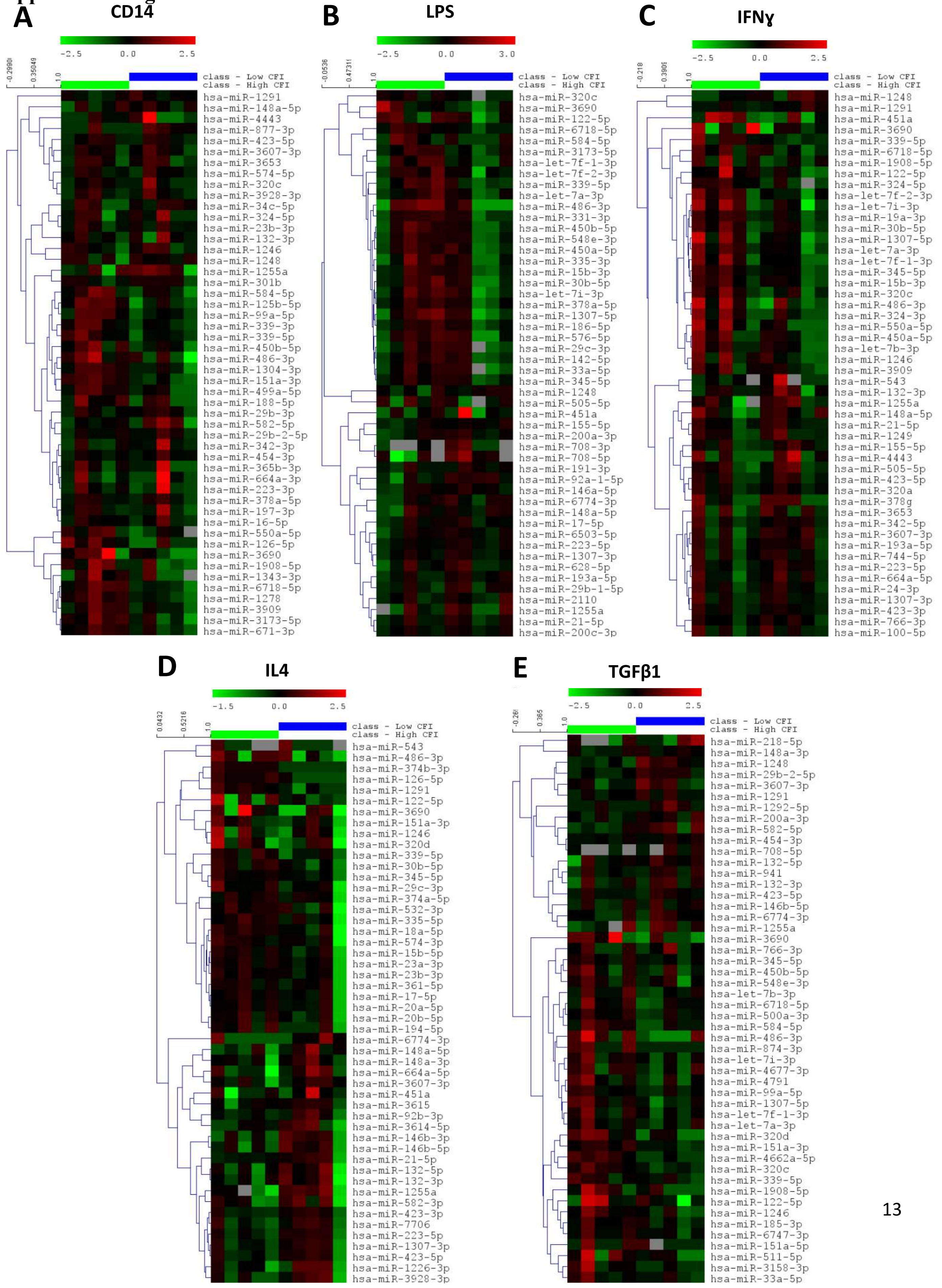

Supplemental Figure 3. Relative extracellular miR339-5p expression shows no differential expression between patients varying capacity of the collateral circulation. Quantitative polymerase chain reaction measurements of miR339-5p in the supernatant of monocyte/macrophages from the entire patient cohort (n = 26) consisting of high versus low collateral capacity. (A) Freshly isolated CD14 monocyte, (B) monocyte cultured without stimulant or monocytes cultured with stimulant (C: LPS, D: IFNɣ, E: IL4, F: TGFβ1). Data are presented as mean ± SD. LPS: Lipopolysaccharide; IFNɣ: Interferon gamma; IL4: Interleukin 4; TGFβ1: Transforming growth factor beta 1.

Supplemental Figure 3.

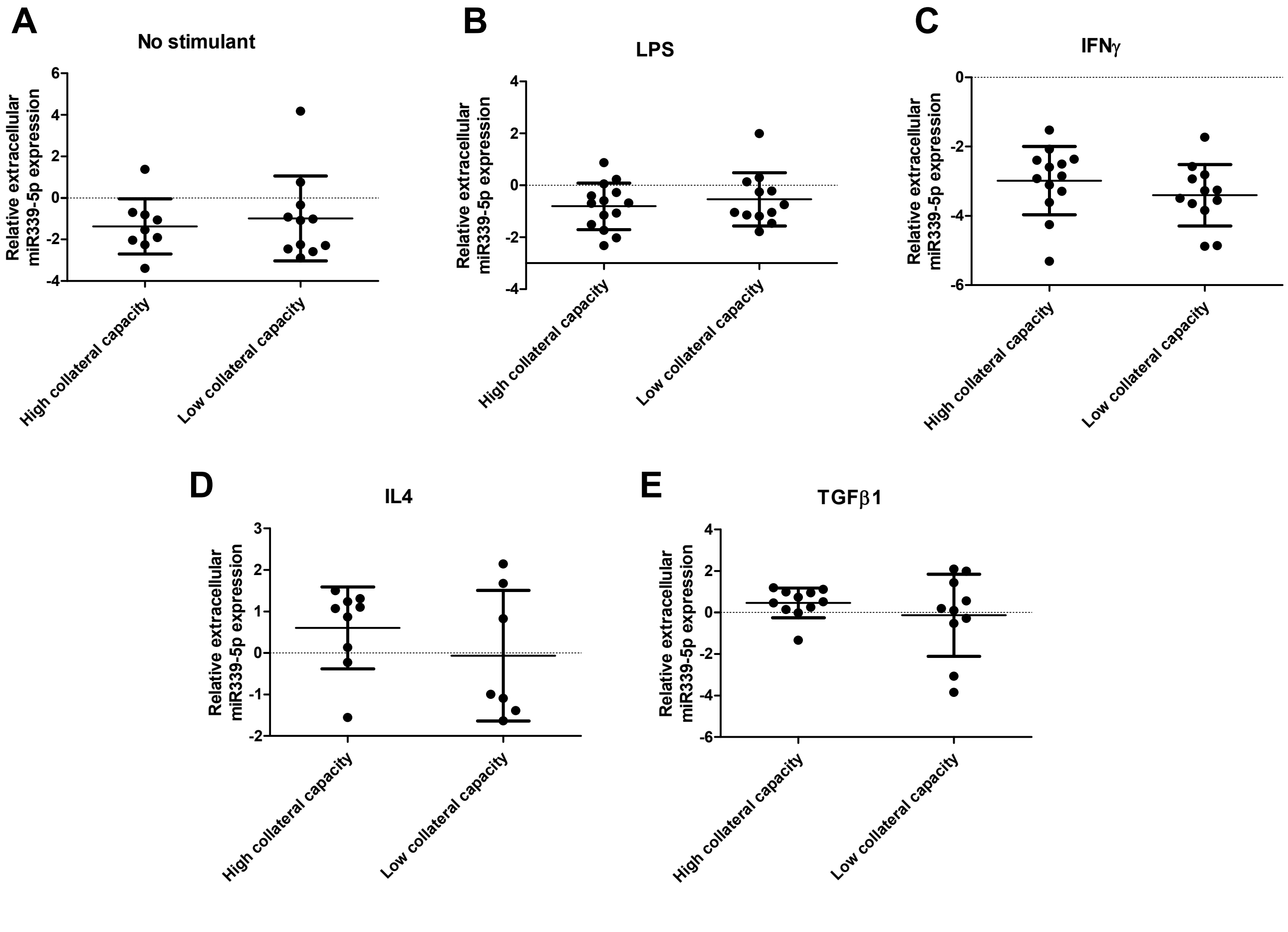

Supplemental Figure 4. Relative cellular miR30b-5p expression is lower in IFNɣ stimulated monocytes of patients with low collateral capacity. Quantitative polymerase chain reaction measurements of miR30b-5p in monocyte/macrophages of the total patient cohort (n = 26) with high versus low collateral capacity. Data are presented as mean ± SD. * $p < 0.05$. IFNɣ: Interferon gamma.

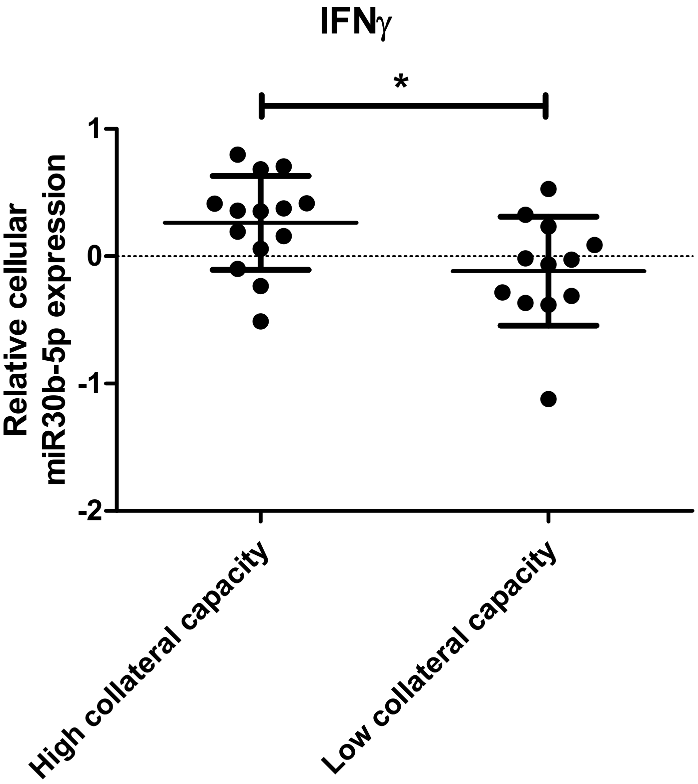

Supplemental Figure 5. STAT3 Pathway depiction using Ingenuity Pathway Analysis (Ingenuity Systems, http://www.ingenuity.com)**.** Ingenuity pathway analysis revealed that predicted gene targets of miR339-5p and CFI_p associated transcripts from LPS and monocytes cultured for 20 hours without stimulant show significant association with members of growth factor receptor family of genes (high-lighted in green). Other respective target molecules within the STAT3 pathway that are associated with miR339-5p gene sets, and CFI_p associated transcripts are outlined in red. BCL2, B-cell lymphoma 2; BCR-ABL, breakpoint cluster region – Abelson fusion protein; CDC25A, cell division cycle 25 homolog A; CIS, cytokine-induced STAT inhibitor; ERK1/2, extracellular signal-regulated protein kinases 1 and 2; GF, growth factor; GRIM-19, gene associated with retinoic-interferon-induced mortality 19; GTP, guanosine-5'-triphosphate; JAK2, janus kinase 2; JNK, c-Jun N-terminal kinase; MKK, mitogen-activated protein kinase kinase; MLK, mixed lineage kinase; PIAS3, E3 SUMO-protein ligase; PIM1, Proto-oncogene serine/threonine-protein kinase; PTP, protein-tyrosine phosphatase; RAC1, Ras-related C3 botulinum toxin substrate 1; SHP1, Src homology region 2 domain-containing phosphatase-1; SOCS*,* Suppressor of cytokine signaling *3;* STAT3*,* Signal transducer and activator of transcription 3; TYK2, tyrosine kinase 2.

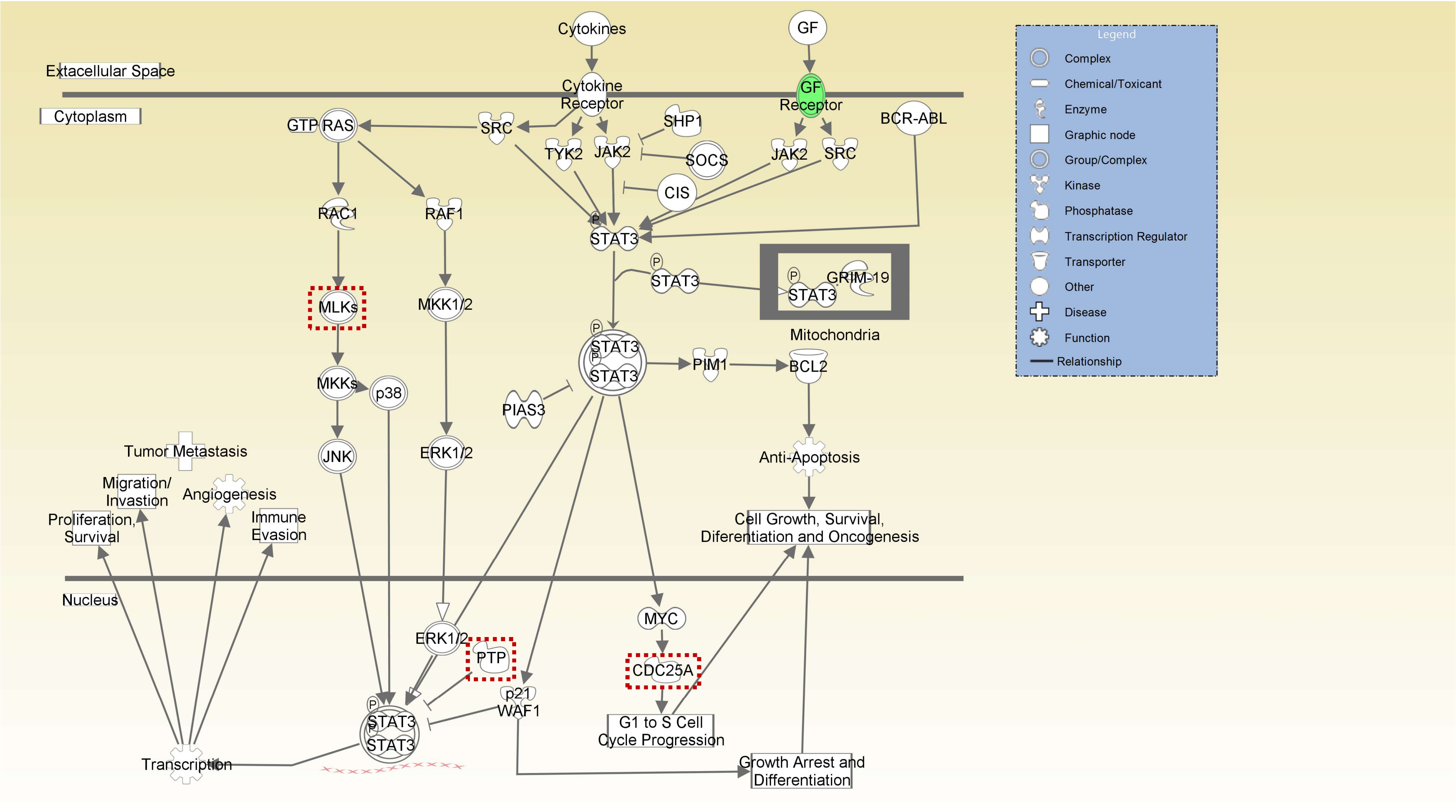

© 2000-2017 QIAGEN. All rights reserved.

Supplemental References

1. Robinson, M.D. & Oshlack, A. A scaling normalization method for differential expression analysis of RNA-seq data. *Genome Biol* **11**, R25 (2010).
